# Pesticide Spraying and Reduced Cholinesterase Activity among Hill Tribe Farmers in Thailand

**DOI:** 10.5696/2156-9614-11.31.210908

**Published:** 2021-08-17

**Authors:** Kowit Nambunmee, Tharinya Kawiya, Richard L Neitzel, Prapamon Seeprasert

**Affiliations:** 1 School of Health Science, Mae Fah Luang University, Chiang Rai, Thailand; 2 Urban Safety Innovation Research Group (USIR), Mae Fah Luang University, Chiang Rai, Thailand; 3 Department of Environmental Health Sciences, School of Public Health, University of Michigan, Michigan, USA

**Keywords:** Lahu hill tribe, farmer health, acetylcholinesterase, language communication, exposure assessment, pesticide spraying

## Abstract

**Background.:**

Farming is an important occupation in Thai hill tribe communities, which are often remote, and lack other economic opportunities along with basic educational, health care, and occupational health and safety services. Additionally, these communities have a unique culture and language.

**Objectives.:**

The present study was conducted in northern Thailand to evaluate pesticide exposures and associated health impacts among hill tribe farmers, and to compare them to Thai farmers.

**Methods.:**

Lahu hill tribe farmers in a mountain community were recruited by public health hospital staff, along with a reference group of lowland Thai farmers. Participants completed a survey on demographic factors and work practices, and blood and urine samples were collected by a trained nurse. Acetylcholinesterase activity (AChE) was quantified to assess pesticide exposure, whereas liver and kidney functions were evaluated using clinical biomarkers.

**Results.:**

A large fraction (nearly 50%) of Lahu farmers were illiterate and could not speak Thai. Thai farmers worked fewer hours per week (39.4) than did Lahu farmers. Among Lahu farmers, AChE levels were significantly lower (worse) than those of Thai farmers. However, other health outcomes in these populations were similar. Formal education and language skills were not associated with pesticide exposures or health outcomes. Pesticide spraying was found to be a significant predictor of reduced AChE (OR=8.5, 95% CI 1.1–69.6).

**Conclusions.:**

Pesticide exposures are a significant occupational health hazard among Thai hill tribe farmers. Training, potentially delivered by community health volunteers, is needed to communicate safe pesticide work practices to these farmers.

**Participant Consent.:**

Obtained

**Ethics Approval.:**

The study protocol was approved by the Institutional Review Board of Mae Fah Luang University (REH-61080).

**Competing Interests.:**

The authors declare no competing financial interests.

## Introduction

Farming is a major occupation and source of family income in Thailand. The northern region of Thailand has excellent conditions for growing high quality crops and has the highest rate of corn cultivation among all regions of Thailand. A total of 793 333 hectares are harvested for corn in upland areas of the region for corn cultivation between May and October.^1^

Pesticides are often used to enhance agricultural production in Thailand; 110 000 tons of pesticides were imported into the country in 2007, which increased to 172 000 tons in 2013.^2^ Most Thai farmers are self-employed or work informally, and pesticide use among informal workers is not regularly monitored.^3^ Although the Thai government has provided guidance to raise awareness of workplace health and safety among self-employed and informal workers, no administrative structure to provide occupational safety and health services to these workers has been established.^4^ Given these circumstances, pesticide use is an ongoing potential occupational risk among farmers.

Acetylcholinesterase activity (AChE) is a marker to indicate exposures to organophosphates (OP) and carbamates (CA), which are the most commonly used families of pesticides.^5^ Lower AChE levels have been associated with OP and CA pesticide exposures.^5^ Measurement of AChE activity can indicate OP and CA exposure before permanent health outcomes manifest,^6^ and can be used to produce estimates of chronic exposure. This biomarker has been used previously in a number of populations, including Thai farmers, a group in which associations were observed between AChE levels and health outcomes.^7^ An important advantage of this biomarker is that it can be processed quickly with basic laboratory equipment, which makes field-based pesticide exposure screening more feasible.

Chiang Rai is a province in northern Thailand located close to the border of Myanmar and Laos. Migrants from these two bordering countries, known as hill tribes, settled in Chiang Rai a century ago.^8^ There are six main hill tribe groups in Chiang Rai: Akha, Lahu, Hmong, Yao, Lisu, and Karen. The Lahu tribe is the second most populous population among hill tribe minorities of Thailand.^9^ The Lahu divide themselves into subgroups, such as the Lahu Na (Black Lahu), Lahu Nyi (Red Lahu), Lahu Hpu (White Lahu), and Lahu Shi (Yellow Lahu); these subgroup names refer to the colors of each subgroup's traditional clothing.^10^ Lahu hill tribes have a unique and distinct culture, and their main occupation is farming (primarily corn, rice, and flowers) in mountainous areas. 9 This activity provides financial support and can be done locally, without the need to travel outside hill tribe villages. However, these villages are commonly located in remote areas, with limited facilities for education, electricity, clean water and health care services. Pesticides are commonly used by Lahu farmers to increase crop yields. The companies selling these pesticides provide manuals and/or guideline labels, but this information is not used by most farmers due to literacy barriers. Limited literacy and lack of formal education may increase pesticide exposure risks,^11^ and awareness of occupational hazards appears to be low in this population. Given that pesticide exposure may represent a significant health hazard among informal workers and minority groups with limited access to education and basic services in Thailand, the present study was conducted to assess health status and the relationship between pesticide exposures and agriculture practices among Lahu farmers in Chiang Rai, and to compare their results to those from a group of Thai farmers.

**Abbreviations**
*AChE*: Acetylcholinesterase activity
*FECa*: Fractional excretion of calcium
*GER*: Glomerular filtration rate
*PPE*: Personal protective equipment

## Methods

**Figure 1:**
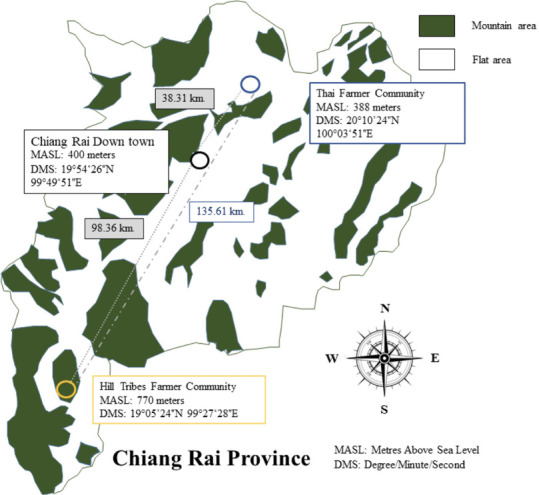
Survey area for Lahu farmers in Ban Huai Muang, Wiang Pa Pao District and Thai farmers in Ban Phan Suek, Mae Chan District

The study protocol was approved by the Institutional Review Board of Mae Fah Luang University (REH-61080). All study participants were 21 years and older and resided in Chiang Rai Province. Staff from the Mae Chedi Mai Health Promoting Hospital helped recruit Lahu farmers in the survey area. The Lahu study community was located nearly 100 km from Chiang Rai in a mountainous area (770 m about sea level) *(Figure 1).* Family information on record at the Mae Chedi Mai Health Promoting Hospital from 2018 was used to identify Lahu farmers to approach for participation; 146 Lahu farmers were identified in this way. One hundred Lahu farmers agree to participate, and Mae Chedi Mai Health Promoting hospital staff then scheduled data and sample collection. A Lahu language translator from the Lahu community was used to interview participants who were not able to speak Thai. A reference group of Thai farmers was recruited by Tha Khao Plueak Health Promoting Hospital staff using the same methods; 86 Thai farmers were identified in this way and approached to participate. The Thai farming community was located more than 130 km away from the Lahu study community in a flat lowland area (mean elevation 388 m above sea level).

### Sample collection

Farmers in the two communities were provided with an overview of the study, and the 100 interested individuals provided informed consent and were enrolled into the study. Individuals unable to provide written consent indicated consent with a thumbprint. All subjects participated in work observations and provided urine and blood samples. To increase community engagement, a community leader and community health volunteer were invited to join the research team during data collection, following consent of the participants.

We assessed pesticide exposures and potential adverse health effects among participating farmers using a combination of survey data, biomarker collection and analysis, and environmental sampling. The survey used was based on a previous study of worker health in a marginalized community *(Supplemental Material).*^12^ The survey was administered at the local community center in the two communities and took 35–45 minutes to complete. Survey questions addressed demographic factors, farm practice, pesticide use, self-reported health and presence of several pesticide-related neurological symptoms, including numbness, tingling, and trembling.

Trained nurses collected 10 mL of urine and 10 mL of venous blood from workers. Five ml of blood was collected in BD Vacutainer^®^ Plus venous blood collection serum tubes, and another 5 ml was collected in BD Vacutainer^®^ EDTA tubes. Serum tubes were left at room temperature for 2 hours to allow blood clotting and then centrifuged at 4 500 rpm for 15 minutes. Serum was separated into cryo-vials. All blood and urine samples were stored at −80°C for further analysis.

### Blood and urine analysis

Urinary creatinine, urinary calcium, serum creatinine, serum calcium, aspartate aminotransferase (AST), and complete blood counts were measured by Meng Rai Laboratory in Chiang Rai, which is accredited by the Medical Technology Council of Thailand. Analyses were completed with an XN-550 Hematology Analyzer (Sysmex Corporation, Kobe, Japan) and COBAS INTEGRA^®^ 400 Plus Analyzer (Roche, Basil, Switzerland). Levels of AChE were analyzed from whole blood using the Ellman method.^13^ Levels <8 200 units/liter (U/L) were classified as reduced AChE activity and presumed high pesticide exposure. Levels >8 200 U/L were classified as normal AChE activity.^14^

### Blood chemistry test

A battery of blood parameters was used to assess health impairment. Anemia was defined in men as hemoglobin (Hb) <13 mg% and in women as Hb <12 mg%. Liver injury was defined as AST >40 U/L. Glomerular filtration rate (GFR) was estimated from serum creatinine levels using the Modification of Diet in Renal Disease (MDRD) equation: GFR <90 ml/min/1.73 m^2^ was defined as kidney impairment.^15^ Fractional excretion of calcium (FECa), the percentage of calcium clearance as a fraction of creatinine clearance, was calculated according to Steward 2012,^16^ and FECa >2% was interpreted as abnormally high excretion of Ca via urine.^17^

### Personal protective equipment use

Self-reported use of personal protective equipment (PPE) was used to group farmers into PPE categories. This method was applied from a 2020 study by Mueangkhiao *et al.*^18^ The PPE-0 category was assigned to farmers who reported no use of PPE (i.e., reported PPE protection percentage = 0). The PPE-1 category was assigned to farmers who reported using face shields, fabric/leather gloves, or other protective clothing (protection percentage = 20). The PPE-2 category was assigned to farmers who reported using cartridge respirators, gas masks, or disposable outer clothing (protection percentage = 30), PPE-3 was assigned to farmers who reported using chemical resistant rubber gloves (protection percentage = 40), and PPE-4 was assigned to farmers who fell into both the PPE-1 and PPE-3 categories (protection percentage = 60).

### Statistical analyses

We calculated summary statistics of survey-derived demographic data across the entire population. We compared distributions of participants according to ethnicity, gender, education, language skills, working hours, farm type, PPE use category, health status, AChE level, neurological symptoms, blood chemistry, and pesticide use. These comparisons were done using chi-square or Fisher's exact tests for categorical variables, and independent Student's t-test for continuous variables. A series of logistic regression models were used to estimate the odds ratio (OR) for reduced levels of AChE (i.e., level <8 200 U/L) associated with different pesticide use activities, neurological symptoms, and blood test results. These models were adjusted for age, ethnicity (i.e., Thai vs. Lahu), PPE category, working hours per week, and education status. All analyses were conducted using the Statistical Package for the Social Sciences (SPSS) program (SPSS, Inc. IBM SPSS, Armonk, NY).

## Results

A total of 100 Lahu farmers participated in the study; one was excluded from data analysis because of kidney disease. Of the 99 remaining Lahu farmers, 95 (96%) were Black Lahu and 4 (4%) were Red Lahu.^10^ Forty-three Thai farmers participated in the study. The proportion of females in the Lahu and Thai groups was similar *(Table 1).* Among the participants, 41.4% of Lahu farmers and 9.3% of Thai farmers were illiterate. Just over 14% of Lahu farmers could not speak Thai, and nearly 50% could not read Thai. A significantly higher fraction (65.1 vs 24.2%, p<0.001) of Thai farmers drank alcohol compared to Lahu farmers, while smoking rates did not differ significantly between the groups (Thai = 27.9, Lahu = 31.3%). There was no identified association between PPE use and reduced levels of AChE among the farmers in the present study.

**Table 1:** Population Characteristics, Farm Practices and Overall Health Status of Surveyed Farmers (N=142)

	Thai (n=43)		Lahu (n=99)		Chi-square p-value

	N	%	N	%	
*Sex*					
Women	23	53.5	50	50.5	
Men	20	46.5	49	49.5	
*Ethnic group*					
Thai	43	100.0	-	-	
Black Lahu	-	-	95	96.0	
Red Lahu	-	-	4	4.0	
*Education*					
Illiterate	4	9.3	41	41.4	<0.001
Primary school	25	58.1	38	38.4	
>=Secondary school	14	32.6	20	20.2	
*Speak Thai*					
Cannot	0	0.0	14	14.1	<0.001*
Fair	1	2.3	42	42.4	
Fluent	42	97.7	43	43.4	
*Read Thai*					
Cannot read	2	4.7	48	48.5	<0.001*
Fair	4	9.3	22	22.2	
Fluent	37	86.1	29	29.3	
*Alcohol use*					
No	15	34.9	75	75.8	<0.001
Yes	28	65.1	24	24.2	
*Smoking*					
No	31	72.1	67	67.7	0.658*
Yes	12	27.9	31	31.3	
*PPE use category*					
PPE-0	4	9.3	7	7.1	0.756*
PPE-1	20	46.5	42	42.4	
PPE-4	19	44.2	50	50.5	

Abbreviation: PPE, personal protective equipment

^*^Fisher exact test p-value

The vast majority of Thai (85%) and Lahu (80%) farmers had worked in this occupation >10 years. A much larger fraction of Lahu farmers grew corn (84.4%) compared to Thai farmers (47.4%). Fewer than 10% of Thai and Lahu farmers used no PPE during farming activities (i.e., were categorized as PPE-0) *(Table 1)*, and roughly half of farmers in the two groups were categorized in the PPE-4 category. Roughly 1–2% of farmers in the two groups reported being in poor health.

Thai farmers had a significantly higher mean age than Lahu farmers (51.2 vs 39.0 years old, p<0.001), but reported fewer working hours per week (39.4 hours vs. 44.0 hours, respectively) *(Table 2).* Lahu farmers had a significantly higher mean hemoglobin level than did Thai farmers (Hb: 14.9 vs 13.6 mg%, respectively, p<0.001), and a significantly lower mean AChE level (10,730.2 vs 13,834.2 U/L, respectively p<0.001).

**Table 2:** Comparison of Age, Working Hours per Week, Acetylcholinesterase Activity, Hemoglobin, Aspartase Aminotransferase, Fractional Excretion of Calcium, and Glomerular Filtration Rate Between Thai and Lahu Farmers (N=142)

	**Thai (n=43)**		**Lahu (n=99)**		T-test p-value

	Mean	S.D.	Mean	S.D.	
Age (years)	51.2	9.0	39.0	12.3	<0.001
Working hours per week	39.4	22.6	44.0	17.9	0.238
AChE (U/L)	13834.2	6003.7	10730.2	4067.2	<0.001
Hb (mg%)	13.6	1.2	14.9	1.5	<0.001
AST (SGOT)	23.1	7.5	23.3	7.3	0.886
FECa(%)	1.5	0.9	0.9	0.6	<0.001
GFR (ml/min/1.73)m^2^	88.0	17.9	94.7	15.7	0.025

Abbreviations: AChE, acetylcholinesterase activity; Hb, hemoglobin; AST, aspartase aminotransferase; FEFCa, fractional excretion of calcium; GFR, glomerular filtration rate.

**Figure 2:**
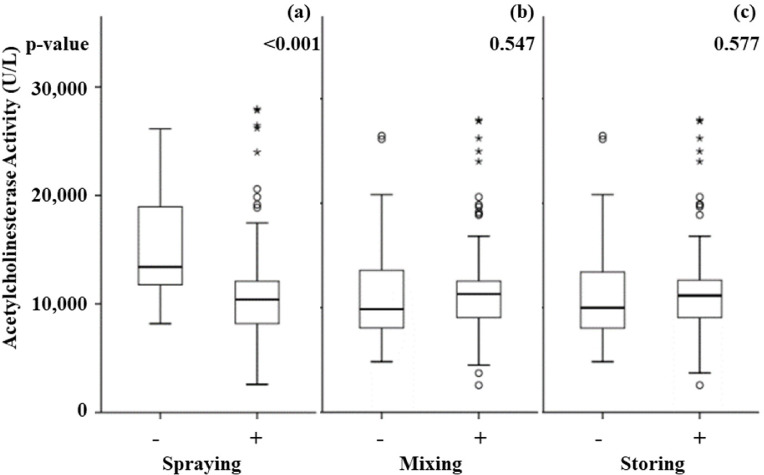
Acetylcholinesterase activity level comparison between groups involved with (+) and not involved with (−) different pesticide use activities

No relationship was observed between reduced AChE activity and language communication or harmful perception of pesticides *(Table 3).* The prevalence of AChE <8 200 U/L among subjects who reported health problems was not significantly different than among participants with no reported health problems. However, AChE <8 200 U/L prevalence was significantly higher among participants with secondary school education than among illiterate subjects (29.4 vs 8.9%, respectively, p<0.028). In addition, mean AChE level among participants with a history of pesticide spraying was significantly lower than that of participants with no history of pesticide spraying *(Figure 2a).* AChE levels did not show a significant relationship with pesticide mixing or storing *(Figure 2b and 2c).*

**Table 3:** Relationship Between Reduced Acetylcholinesterase (AChE) Activity, Language Skills, Education, Pesticide Danger Perception, and Self-reported Health Problems in Thai and Lahu Farmers (N=142)

	AChE (Unit/L)				Chi-square p-value

	>8,200		<8,200		
*Speak Thai*					
Cannot	12	85.7	2	14.3	0.717*
Fair	32	74.4	11	25.6	
Fluent	67	78.8	18	21.2	
*Read Thai*					
Cannot read	42	84.0	8	16.0	0.326
Fair	18	69.2	8	30.8	
Fluent	51	77.3	15	22.7	
*Education*					
Illiterate	41	91.1	4	8.9	0.028*
Primary school	46	73.0	17	27.0	
>=Secondary	24	70.6	10	29.4	
*Perceive harm*					
No	28	82.4	6	17.7	0.372
Yes	62	74.7	21	25.3	
*Health problems*					
No	52	77.6	15	22.4	0.879
Yes	59	78.7	16	21.3	

Abbreviation: AChE, acetylcholinesterase activity.

^*^Fisher exact test p-value

As shown in Table 4, logistic regression was used to calculate the odds ratio (OR) comparing reduced AChE prevalence between presence and absence of neurological symptoms, blood chemistry test results and pesticide use. A subject with a history of pesticide spraying had a 8.546 times chance of having reduced AChE (1.050–69.557 95% C.I., p=0.045) after adjusting for age, ethnic group, PPE category, working hours per week and education. A relationship between tingling and reduced AChE was not found in this analysis, which implies the presence of a confounding factor in this relationship. No relationship between liver or kidney injury and reduced AChE activity was found in this analysis.

**Table 4:** Logistic Regression Analysis to Assess Risk of Reduced Levels of Acetylcholinesterase Activity (<8,200 U/L) Associated with Neurological Symptoms, Blood Chemistry, and Pesticide Use ^*^

	Adjusted Odds	Lower CI	Upper CI	p-value
***Neurological symptom***				
Numbness	0.57	0.23	1.42	0.228
Trembling	0.48	0.12	1.89	0.295
Tingling	0.32	0.09	1.18	0.087
***Blood chemistry test***				
Anemia	1.55	0.27	8.91	0.626
AST (>40 U/L)	0.56	0.06	5.36	0.618
FECa (>2.00%)	1.42	0.37	5.51	0.615
GFR (<90 ml/min/1.73m^2^)	1.17	0.42	3.24	0.767
***Pesticide using***				
Spraying	8.55	1.05	69.56	0.045
Mixing	0.54	0.22	1.32	0.177
Storing	0.39	0.16	0.98	0.044

Abbreviations: AST, aspartate aminotransferase; CI, confidence interval; FECa, fractional excretion of calcium.

^*^All models adjusted for age, ethnic group, PPE category, working hours and education level.

## Discussion

In 2012, there were 216 hill-tribe villages with a population of 48 835 in Chiang Rai province.^9^ This population has limited access to education and other services due to their mountainous location and cultural differences, and these conditions result in increased health risks, including risk of exposure to occupational hazards. Most hill tribe farmers are informal workers who do not have the infrastructure and resources necessary to create healthy workplace environments.

Exposures to pesticides are a major occupational hazard for farmers, and can occur during storing, mixing and spraying activities.^19^ Health outcomes associated with acute pesticide exposure include, dizziness, cramps, nausea, vomiting, abdominal pain, numbness, fatigue, headache, excessive salivation, respiratory problems and blurred vision.^20^ Chronic health outcomes resulting from pesticide exposure include kidney and liver dysfunction and increased cancer risk.^18^ In Thailand, the rate of pesticide-related morbidity increased from 2.5 to 9.0 per 100 000 population from 2010 to 2015, with 70.8% of cases occurring in individuals between the ages of 15 and 59.^21^ This situation suggests that safe practical guidelines are needed to reduce pesticide exposures among farmers in Thailand.

The present study evaluated pesticide-related activities and exposures as well as health outcomes in Lahu farmers compared to Thai farmers. While poverty and poor health are often more common in marginalized communities, roughly 10% of participating Thai and Lahu farmers had expenses that exceeded their income, and the majority of farmers in both groups reported good health. Despite these similarities, differences in blood chemistry were found between the two groups. In Thai farmers, AChE levels were significantly higher than Lahu farmers, reflecting differences in pesticide exposure levels. Two measures of kidney function, FECa and GFR, were significantly different between the Thai and Lahu farmers. Age may be one explanation for this finding, given the significantly higher mean age of the Thai farmers, therefore logistic regression models were used to adjust for age in the present study. We did not find differences in the prevalence of renal and liver dysfunction, evaluated as presence increased AST, increased FECa, or decreased GFR among participants with reduced AChE compared to those with normal AChE. This indicates that pesticide exposures did not appear to contribute to more health impairments. A similar result was reported in a study by Aroonvilairat *et al.*,^7^ in which the prevalence of health impairment did not differ significantly between pesticide-exposed and non-exposed farmers. On the other hand, reduced AChE prevalence was significantly higher (adjusted OR of 8.55, Table 4) in pesticide spraying farmers compare to non-spraying farmers, showing that AChE reflected pesticide exposure more than health impairment.

Farmers can be exposed to pesticides via inhalation, skin absorption, and ingestion resulting from three basic activities: mixing and loading, spraying, and storing. In the present study, only pesticide spraying was significantly related to reduced AChE. The prevalence of AChE <8 200 U/L (indicating reduced AChE) was six times higher (25.6%) for farmers who sprayed pesticides compared to those who did not (4.0%). There was an 8.5 (95% CI 1.05–69.6) OR for reduced AChE among farmers who sprayed pesticides, adjusting for age, ethnicity, PPE category, working hours per week, and education status. Spraying may result in a longer exposure duration compared to other pesticide-related activities, which may result in greater doses of pesticides.^22^ Other studies have reported a substantial body burden of pesticides among pesticide sprayers.^23^

Pesticide spraying increases pesticide exposure via inhalation and dermal absorption. Dermal absorption may occur as a result of splashes, spills, or spray drift, whereas small droplets can be inhaled.^23^ Kokkinaki *et al.*^24^ showed that pesticide metabolite levels in urine samples from pesticide sprayers were higher than in a control group. This indicates a higher pesticide exposure risk among sprayers. Contributing factors for pesticide exposure from spraying include spraying duration and frequency, smoking, wind direction, temperature, pesticide type,^25^ spray nozzle type,^26^ spraying equipment cleaning, and PPE use.^27^

**Figure 3:**
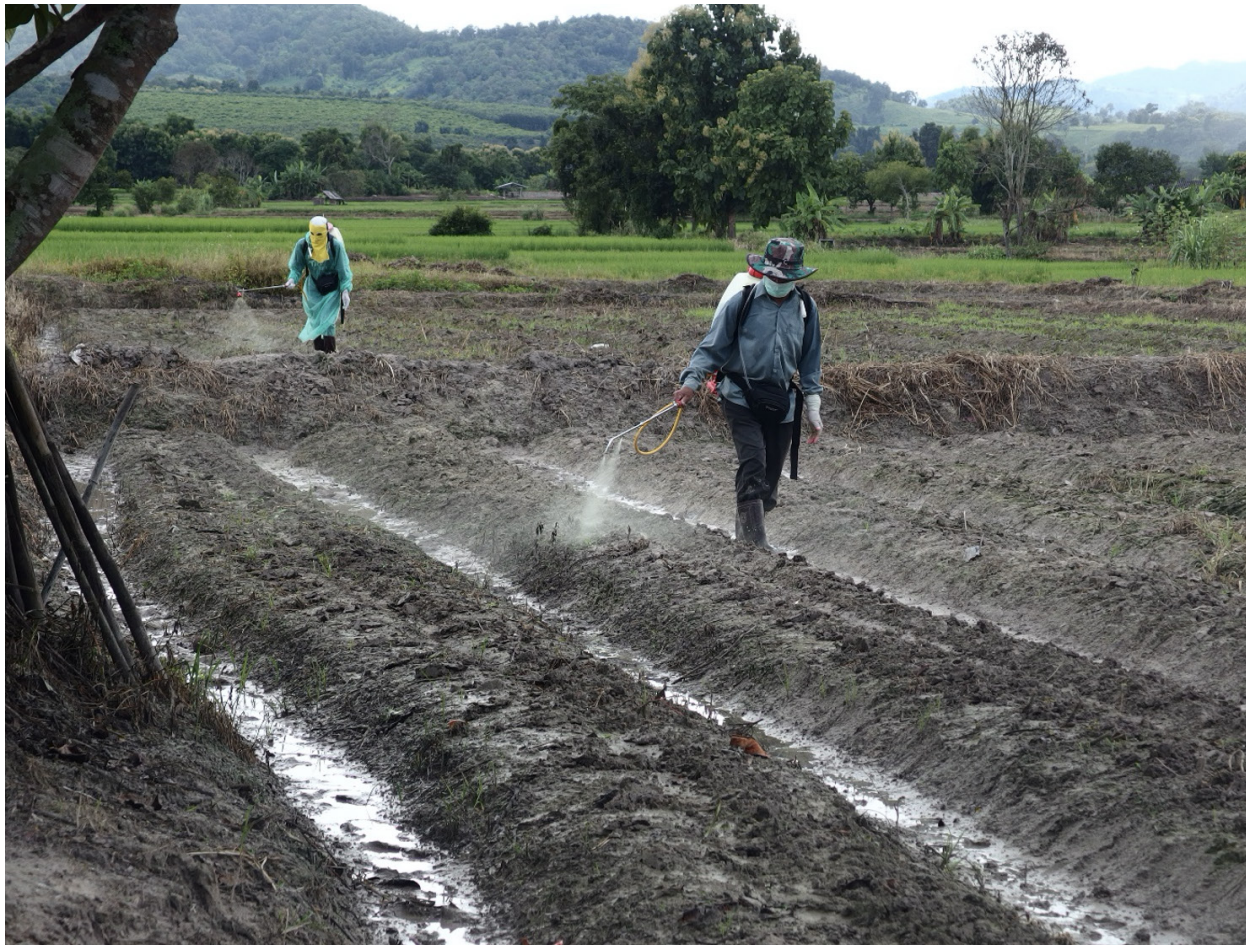
Thai farmer using personal protective equipment during pesticide spraying

Roughly half of Thai and Lahu farmers in this study were assessed to have minimal use of PPE (i.e., categories PPE 0 or PPE 1). Personal protective equipment use in this population, and particularly among farmers in the remote Lahu community, may be limited due to factors such as discomfort, weight, and restriction of movement,^28^ in addition to potentially reduced access to PPE due to lack of availability or inability to afford, which have previously been documented as barriers to use by Satya *et al.*^29^ Figure 3 shows common pesticide spraying behavior among both Thai and Lahu farmers, which include lack of chemical resistant gloves and use of a surgical mask with a cloth cover as PPE. This type of face covering is not recommended for handling pesticides,^30^ and is not proper respiratory protection for pesticide application. Additionally, rather than relying on PPE for protection, the hierarchy of controls dictates that engineering and administrative controls should be implemented to reduce pesticide exposure. Such controls might include substitution with less toxic pesticides, improved designs for the control of spray direction and timing, and worker training.

The present study found no relationship between formal education, Thai language skills, and literacy and reduced levels of AChE. Okoffo *et al.*^31^ suggested that the lower level of education of these farmers may affect their work behaviors and subsequent health status.^31^ However, Mattah *et al.*^32^ suggested that formal education may not impact farmer work practices and exposures differently than informal training.^32^ Such informal training could include discussions with pesticide retailers, other farmers, community leaders with knowledge regarding farming techniques,^29^ and pesticide packaging and marketing materials. Based on the experience of the research team, the latter contained information that was difficult to apply in real farm-working scenarios. Retailers and other farmers therefore represent key sources of information regarding the safe application of pesticides by farmers. Such individuals may practice farming using similar practices, come from the same community, and share culture and language.^33^ Informal discussions with these information sources could occur during routine farm activities or community recreational activities. The consistency and accuracy of knowledge transferred to farmers by pesticide retailers and other farmers may not be sufficient to ensure adoption of proper practices; nevertheless, this does represent a useful means of farmer education.

Community health volunteers may represent a reliable mechanism for communicating pesticide safety information to informal farmers. In Thailand, health volunteers are villagers who are paid by the Thai government to promote public health in the community.^34^ Since these volunteers come from local villages, they understand the culture and lifestyle of people in those villages. The volunteers work with the local hospital to perform community health monitoring for vital health issues and provide basic treatments. They have a reputation in Thailand as an accurate and practical source of health information. If safe pesticide farming practices and information could be introduced to health volunteers, they could then serve as a key resource to famers, and provide on-the-job training, work practice observation, and farmer education.

Any efforts to promote pesticide safety among farmers must consider economic factors. Farmers often focus on income more than health due to the need to financially support their families. Health outcomes caused by chronic occupational pesticide exposure develop slowly over time, making them a lower priority than immediate economic needs. Lekei *et al.*^33^ mentioned that farmers experiencing pesticide poisoning may not seek medical treatment for a number of reasons, including inability to afford payment for medical bills, the mild severity of most poisoning cases, anticipated difficulty in diagnosis and treatment, distance to nearest health care facility or poor access to health service; and lack of appropriate medical treatment options. In Thailand, universal health care provides coverage for pesticide poisoning treatment and pesticide exposure screening, but accessibility of health services is critical issue for remote communities such as the Lahu farming community assessed in the present work. A sensitive, simple, and easily administered and evaluated biomarker to examine early exposures and health risks could help resolve this situation.

AChE represents a suitable biomarker to screen pesticide exposure, perhaps through the use of mobile clinics during the agricultural season. The biomarker only indicates exposure to organophosphate and carbamate insecticides, but can nevertheless provide very useful information to farmers, particularly given the prevalence of these pesticides in Thailand. The benefits of AChE include a short processing time and cost effective method of assessing pesticide exposure. Widespread use of this biomarker in Thai and hill tribe farming communities could identify cases of early, mild pesticide intoxication and guide both prevention and treatment of more severe intoxication.

### Limitations

Language barriers were a major limitation of the present study; interviews took longer to conduct among Lahu farmers compared to Thai farmers. This could introduce differences in response accuracy between the two groups for questions regarding PPE use, overall health, perceptions of harm and neurological symptoms. We were not able to compare AChE levels before and after the spraying season; that approach would have allowed us to assess the impact of spraying on pesticide accumulation in farmers. Additionally, more details on spraying activities would have been useful, including frequency and duration of spraying, spraying nozzle type, spray timing, and spraying tool maintenance and cleaning methods.

## Conclusions

The Lahu farmers in the present study were informal workers with limited access to public and occupational health services and experienced pesticide exposures. This study highlights the utility of mixed-method evaluation of pesticide-related farming activities, exposures, and health impacts, and the results can help guide practical recommendations for pesticide exposure reduction. Our results suggest that training is needed to support safe pesticide practices. Such training could be implemented at the beginning of the planting season through community health volunteers, who can effectively communicate this information to Lahu farmers in their native language. Any training should be complemented by health services to monitor pesticide exposures and promote healthy lifestyles. The research should be expanded to include other countries in Southeast Asia that have hill tribe communities to evaluate the degree to which pesticide exposure risks differ by country, and to assess whether similar pesticide exposure reduction strategies might be viable across these countries. Nevertheless, AChE is an acceptable biomarker that is inexpensive and readily available. Future research should evaluate pesticide exposures in a more controlled manner, with measurement immediately before and after spraying activities, which would allow for greater precision in assessing the association between exposures and health outcomes.
