# Supplementary material for: Pesticide Spraying and Reduced Cholinesterase Activity among Hill Tribe Farmers in Thailand
Source: J Health Pollut. 2021 Aug 17;11(31):210908. doi: 10.5696/2156-9614-11.31.210908 (PMC8383788; doi:10.5696/2156-9614-11.31.210908)
Supplement: Supplementary file 1 [file Nambunmee_Supplemental_Material.docx]

**Supplemental Material**

| **Part 1: Demographics** |
| --- |

***Now I will ask you some questions about yourself.***

1. **What gender do you currently identify as?** ☐_1_ Male ☐_2_ Female ☐_3_ Other
2. **What was your age at your last birthday?** ____________years
3. **Do you live in Chiang Rai?**

☐_1_ Yes ☐_0_ No ☐_888_ Don't know

1. **What is your ethnic group?**

☐_1_ Thai ☐_2_ Red Lahu ☐_3_ Black Lahu ☐_4_ White Lahu ☐_5_ Yellow Lahu

☐_6_ Others (please specify)_______________ ☐_888_ Don't know

1. **If yes, how long have you been living in Chiang Rai?** ____________years ____________months
2. **How well do you SPEAK Thai?**

☐_1_ Cannot ☐_2_ Fair ☐_3_ Fluent

1. **How well do you READ Thai?**

☐_1_ Cannot ☐_2_ Fair ☐_3_ Fluent

1. **What is your total household income per month?**

☐_1_ Less than 5000 baht ☐_2_ 5001 - 10000 baht ☐_3_ 10101 - 15000 baht

☐_4_ 15001 - 20000 baht ☐_5_ 20001 - 25000 baht ☐ _6_ 25001 - 30000 baht

☐ _7_ 30001 - 35000 baht ☐ _8_ More than 35000 baht ☐_666_ Prefer not to answer

1. **What is your total household expenditure per month?**

☐_1_ Less than 5000 baht ☐_2_ 5001 - 10000 baht ☐_3_ 10101 - 15000 baht

☐_4_ 15001- 20000 baht ☐_5_ 20001 - 25000 baht ☐ _6_ 25001 - 30000 baht

☐ _7_ 30001-35000 baht ☐ _8_ More than 35000 baht ☐_666_ Prefer not to answer

1. **What are your household’s sources of income:**

| **a. Agriculture?** | ☐_2_Primary income source | ☐_1_Secondary income source | ☐_0_Not a source of income |
| --- | --- | --- | --- |
| **c. Construction/Labor/Blue collar?** | ☐_2_Primary income source | ☐_1_Secondary income source | ☐_0_Not a source of income |
| **d. Textiles/Artisan goods?** | ☐_2_Primary income source | ☐_1_Secondary income source | ☐_0_Not a source of income |
| **e. Professional (Nurse/Teacher, etc.)?** | ☐_2_Primary income source | ☐_1_Secondary income source | ☐_0_Not a source of income |
| **f. Other:___________________?** | ☐_2_Primary income source | ☐_1_Secondary income source | ☐_0_Not a source of income |

1. **What is your marital status?**

☐_1_ Single ☐_2_ Married ☐_3_ Divorced

☐_4_ Living with partner ☐_5_ Widowed ☐_6_ Separated

1. **What is the highest level of school you attended?**

☐_0_ None ☐_1_ Primary ☐_2_ Secondary

☐_3_Some college ☐_4_ Bachelor ☐_4_ Higher

| **Part 2: Work History** |
| --- |

***Now I will ask you some questions about your work history.***

1. **Are you currently working?** ☐_1_ Yes ☐_0_ No
2. **Which of the following jobs is your primary job? (Select only one)**

☐_1_ Farming/agricultural work ☐_2_ Artisan (Mason/Carpenter/Electrician/Plumber)

☐_3_ Mechanic ☐_4_ Shop owner/Retailer

☐_5_ Trading ☐_6_ General employee

☐_7_ Food vendor ☐_8_ Taxi/Minibus/Truck Driver

☐_9_ Factory/Industry/Private sector worker ☐_10_ Government staff

☐_11_ Retired ☐_12_ Keeping house/Caring for children

☐_13_ School/Full time study ☐_777_Other: _____________________

1. **If you have other jobs, list them here:** _______________________________________________________________
2. **How long have you worked at your primary job?** ____________years _____________months
3. **What was your previous job? ______________________________ *[Mark “none” if no previous job]***
4. **What years did you begin and end your previous job? Start year:_____________ Stop year:______________**

| **Part 3: Health** |
| --- |

***Now I will ask you some questions about your health.***

1. **How often do you use chemicals to keep insects like mosquitoes away from you at HOME?**

☐_1_ Rarely or never ☐_2_ Occasionally ☐_3_ Always or frequently

1. **How often do you use chemicals to keep insects like mosquitoes away from you at WORK?**

☐_1_ Rarely or never ☐_2_ Occasionally ☐_3_ Always or frequently

1. **In general, my overall health is:**

☐_1_ Poor ☐_2_ Fair ☐_3_ Good ☐_4_ Very Good ☐_5_ Excellent

1. **Do you have any health impairments or health problems that limit the kind or amount of work that you can do?**

☐_1_Yes ☐_0_No

**If yes, please describe:**____________________________________________________________________

1. **In the last two weeks, how often have you had the following conditions?**

| **a. Skin rashes** | ☐_1_ Rarely or never | ☐_2_ Occasionally or usually |
| --- | --- | --- |
| **b. Headache or dizziness** | ☐_1_ Rarely or never | ☐_2_ Occasionally or usually |
| **c.** **Blood in your urine** | ☐_1_ Rarely or never | ☐_2_ Occasionally or usually |
| **d. Blood in your stool** | ☐_1_ Rarely or never | ☐_2_ Occasionally or usually |
| **e. Cough, shortness of breath, or difficulty breathing** | ☐_1_ Rarely or never | ☐_2_ Occasionally or usually |
| **f. Heart beating abnormally** | ☐_1_ Rarely or never | ☐_2_ Occasionally or usually |
| **g. Loose or watery stools** | ☐_1_ Rarely or never | ☐_2_ Occasionally or usually |
| **h. Fever** | ☐_1_ Rarely or never | ☐_2_ Occasionally or usually |
| **i. Nausea or stomach ache** | ☐_1_ Rarely or never | ☐_2_ Occasionally or usually |
| **j. Vomiting** | ☐_1_ Rarely or never | ☐_2_ Occasionally or usually |
| **k. Abdominal pain and cramping (non-menstrual)** | ☐_1_ Rarely or never | ☐_2_ Occasionally or usually |
| **l. Muscle pain** | ☐_1_ Rarely or never | ☐_2_ Occasionally or usually |
| **m. Tingling in toes, fingers, or limbs** | ☐_1_ Rarely or never | ☐_2_ Occasionally or usually |
| **n. Trembling** | ☐_1_ Rarely or never | ☐_2_ Occasionally or usually |
| **o. Numbness** | ☐_1_ Rarely or never | ☐_2_ Occasionally or usually |
| **MORE HERE ………………………….** | ☐_1_ Rarely or never | ☐_2_ Occasionally or usually |

1. **Have you ever been told by a doctor or health professional that you have any of the following medical conditions?**

☐_1_ High blood pressure ☐_2_ Diabetes mellitus ☐_3_ Asthma

☐_4_ Heart disease ☐_5_ Stroke ☐_6_ Kidney disease

☐_7_ Colon cancer ☐_8_ Rectal cancer ☐_9_ Stomach cancer

☐_10_ Liver disease ☐_789_ NA/None of these ☐_777_ Other:_____________________

1. **Are you taking medicine for any of these conditions?**

☐_1_ Yes ☐_0_ No ☐_789_ N/A ☐_888_ Don't know

| **Part 4: Agriculture Work** |
| --- |

***Now I will ask you some questions related to farm work.***

1. **How far is your home from the nearest area where pesticides are MIXED?**

☐_1_ No pesticides mixed on farm ☐_2_ Within the home ☐_3_ Less than 50 meters

☐_4_ 50-100 meters ☐_5_ More than 100 meters

1. **Have you ever been involved in farm work?** ☐_1_ Yes ☐_0_ No
2. **Have you ever been involved in farm work outside of Thailand?**  ☐_1_ Yes ☐_0_ No
3. **Over the last three years, where are your living quarters located?**

| **a. Off farm on property NOT owned or administered by present employer** | ☐_0_ Never | ☐_1_ Almost never | ☐_2_ Sometimes | ☐_3_ Fairly often | ☐_4_ Always |
| --- | --- | --- | --- | --- | --- |
| **b. Off farm on property owned or administered by present employer** | ☐_0_ Never | ☐_1_ Almost never | ☐_2_ Sometimes | ☐_3_ Fairly often | ☐_4_ Always |
| **c. On farm of the grower you currently work for** | ☐_0_ Never | ☐_1_ Almost never | ☐_2_ Sometimes | ☐_3_ Fairly often | ☐_4_ Always |
| **d. Other _________________________** | ☐_0_ Never | ☐_1_ Almost never | ☐_2_ Sometimes | ☐_3_ Fairly often | ☐_4_ Always |

1. **Over your lifetime, how many years have you lived or worked on a farm?**

☐_1_ Less than 5 years ☐_2_ 5-10 years ☐_3_ 11-20 years

☐_4_ 21-30 years ☐_5_ Over 30 years

1. **What are the top three type of farms you have worked on previously? (Choose only three)**

☐_1_ Eggplant ☐_2_ Rubber trees ☐_3_ Rice

☐_4_ Mangoes ☐_5_ Pineapple ☐_6_ Corn

☐_7_ Sugar ☐_8_ Cassava ☐_9_ Flowers

☐_10_Tapioca ☐_11_ Soybeans ☐_12_ Sericulture

☐_13_ Fish ☐_14_ Dairy cows ☐_15_ Other livestock

☐_16_ Other grains ☐_777_ Other:__________________________________

1. **What is the average number of hours you work in a DAY**? ______________ hours
2. **What is the average number of hours you work in a WEEK**? _____________hours
3. **What is the average number of WEEKS you work in a YEAR**? ______________ weeks
4. **In the past three months, how often have you performed the following activities:**

| **a. Sprayed pesticides?** | ☐_0_ Never | ☐_1_ Yes |
| --- | --- | --- |
| **b. Mixed pesticides**? | ☐_0_ Never | ☐_1_ Yes |
| **c. Stored pesticides?** | ☐_0_ Never | ☐_1_ Yes |

1. **What application methods do you generally use when you apply pesticides (insecticides, herbicides)?**

☐_1_ Do not usually apply ☐_2_ Airblast ☐_3_ Boom on tractor, truck, or trailer

☐_4_ Hand spray gun ☐_5_ Backpack sprayer ☐_6_ Aerial (aircraft application)

☐_7_ In furrow or banded ☐_8_ Mist blower/fogger ☐_9_ Other __________________

1. **What types of protective equipment do you use when you personally handle pesticides?**

☐_1_ Never use protective equipment

| 1. **Hat** | ☐_0_ Never | ☐_1_ Yes |
| --- | --- | --- |
| 1. **Goggles** | ☐_0_ Never | ☐_1_ Yes |
| 1. **Eye glasses (prescription or sun)** | ☐_0_ Never | ☐_1_ Yes |
| 1. **Chemical resistant gloves (like neoprene or nitrile gloves)** | ☐_0_ Never | ☐_1_ Yes |
| 1. **Fabric/leather gloves** | ☐_0_ Never | ☐_1_ Yes |
| 1. **Apron** | ☐_0_ Never | ☐_1_ Yes |
| 1. **Chemical resistant boots** | ☐_0_ Never | ☐_1_ Yes |
| 1. **Closed toe shoes** | ☐_0_ Never | ☐_1_ Yes |
| 1. **Cloth coveralls (complete suit)** | ☐_0_ Never | ☐_1_ Yes |
| 1. **Cartridge respirator, gas mask** | ☐_0_ Never | ☐_1_ Yes |
| 1. **Medical/surgical mask** | ☐_0_ Never | ☐_1_ Yes |
| 1. **Full face shield** | ☐_0_ Never | ☐_1_ Yes |
| 1. **Cloth cover face** | ☐_0_ Never | ☐_1_ Yes |
| 1. **Disposable outer clothing** | ☐_0_ Never | ☐_1_ Yes |

☐_9_ Other ___________________________

1. **After mixing or applying pesticides, when do you usually change into clean work clothes?**

☐_1_ Right Away ☐_2_ At lunch

☐_3_ At the end of that work day ☐_4_ At the end of the next work day

☐_5_ Later in the week ☐_6_ Do not clean my work clothes

☐_6_ Always use disposable outer clothing ☐_7_ Other __________________

1. **In your household, how are clothes usually washed after they have been worn when mixing or applying pesticides?**

☐_1_ Always wear disposable clothing ☐_2_ Mixed with family wash

☐_3_ Soaked separately then mixed with family wash ☐_4_ Washed separately but in the family washing area

☐_5_ Sent out or washed in machine used only for this purpose

☐_6_ Don’t Know ☐_7_ Other __________________

1. **After mixing or applying pesticides, where do you usually wash up or shower?**

☐_1_ Bathroom in home ☐_2_ Outside shower ☐_3_ Other area outside home

☐_4_ Don’t know ☐_5_ Don’t wash after ☐_6_ Other __________________

1. **How often do you know what the chemicals you are spraying when you spray pesticides at work?**

☐_1_ Rarely or never ☐_2_Occasionally ☐_3_ Always or frequently

1. **Do you feel like spraying chemicals during work is harmful to your health?**

☐_1_ Yes ☐_0_ No ☐_888_ Don't know

1. **If yes to previous question, please explain:** _______________________________________________☐_789_ NA

| **Part 5: Tobacco and Alcohol Use** |
| --- |

***Now I will ask you some questions about your tobacco and alcohol use.***

1. **Have you smoked at least 100 cigarettes during your entire life (equivalent to about 5 packs)?**

☐_1_ Yes ☐_0_ No ☐_888_ Don’t know ☐_999_ Prefer not to answer

1. **Do you smoke cigarettes now?**

☐_1_ Yes ☐_0_ No ☐_888_ Don’t know ☐_999_ Prefer not to answer

1. **How many cigarettes do you smoke per day?**____________cigarettes
2. **How many days per week do you consume alcohol?**

☐_0_ Never ☐_1_ 1-3 days ☐_2_ 4-6 days ☐_3_ Daily

☐_888_ Don’t know ☐_999_ Prefer not to answer

Notes:______________________________________________________________________________________
